# Real-time imaging of pulvinus bending in *Mimosa pudica*

**DOI:** 10.1038/srep06466

**Published:** 2014-09-25

**Authors:** Kahye Song, Eunseop Yeom, Sang Joon Lee

**Affiliations:** 1 School of Interdisciplinary Bioscience and Bioengineering, Pohang University of Science and Technology (POSTECH), 77 Cheongam-Ro, Nam-Gu, Pohang, Gyeongbuk, 790-784, Korea; 2 Department of Mechanical Engineering, Pohang University of Science and Technology(POSTECH), 77 Cheongam-Ro, Nam-Gu, Pohang, Gyeongbuk, 790-784, Korea

## Abstract

*Mimosa pudica* is a plant that rapidly shrinks its body in response to external stimuli. *M. pudica* does not perform merely simple movements, but exhibits a variety of movements that quickly change depending on the type of stimuli. Previous studies have investigated the motile mechanism of the plants from a biochemical perspective. However, an interdisciplinary study on the structural characteristics of *M. pudica* should be accompanied by biophysical research to explain the principles underlying such movements. In this study, the structural characteristics and seismonastic reactions of *M. pudica* were experimentally investigated using advanced bio-imaging techniques. The results show that the key factors for the flexible movements by the pulvinus are the following: bendable xylem bundle, expandable/shrinkable epidermis, tiny wrinkles for surface modification, and a xylem vessel network for efficient water transport. This study provides new insight for better understanding the *M. pudica* motile mechanism through structural modification.

Plants are generally considered to be non-motile organisms. However, certain plants move in response to environmental changes. For example, wheat awns perform a swirling motion to thrust their seed into the ground^1^, and seed-bearing pine cones open to spread their seeds in response to relative humidity^2^. The biophysical characteristics of these types of plants have also been investigated to classify the hydraulic movements in plants and fungi^3^.

In addition, certain species exhibit dynamic movements for a short period. After bunchberry flowers burst open, their petals rapidly separate and then flip back to release stamens^4^. A Venus flytrap rapidly closes its leaves to catch insects. As a typical movable plant, *M. pudica* exhibits a rapid response to stimuli. Among the multicellular movements in plants and fungi, *M. pudica* drives the most rapid hydraulic motion^3^. The movements duration (about 1 s) is similar to the poroelastic time τ_p_ which determines the most rapid water swelling motion (for a pulvinus with a 200 μm radius, the poroelastic time is τ_p_ ~ 1 s)^5^.

The best-known *M. pudica* motion consists of its leaves quickly closing up and the pulvinus rapidly bending downward. In fact, *M. pudica* exhibits different responses to stimuli, including opening or closing leaves and bending its pulvinus. The pulvinus is a joint-like bulging structure that connects the main stem and petiole, and, it is sufficiently flexible to bend in all directions, which implies that the *M. pudica* pulvinus can move the petiole omnidirectionally.

Then what occurs in the pulvinus? In most previous studies, the *M. pudica* responses were studied from a biochemical perspective. Different osmotic swelling/shrinking phenomena^6^, potassium and chloride ion effluxes^7^,^8^, and sucrose unloading in phloem vessels^9^ reportedly induce a volume change in motor cells^10^ and turgor pressure loss. Furthermore, aquaporins involve motion and facilitate the rate of membrane water transport^11^. In contrast, the water-refilling process is mainly induced through ATPase activity^12^,^13^, which facilitates water transport across vacuolar membranes^14^. These biochemical studies have contributed to our understanding of the *M. pudica* motile mechanism. However, these cellular mechanisms should be supplemented with interdisciplinary research to further elucidate the motile mechanism.

Recently, novel approaches from a morphological perspective have been suggested as a means of understanding plant motile mechanism. For example, the Venus flytrap closure mechanism was structurally investigated; this mechanism is similar to the buckling of an elastic shell, and snap-buckling instability is involved in the closure mechanism^15^. The structural variation of a hydro-actuated ice plant seed capsules was investigated, and actuating a swellable cellulose layer in the plant cells explains the flexing-and-packing mechanism^16^. Herein, we studied the morphological characteristics of the *M. pudica* pulvinus' flexible movements using advanced bio-imaging techniques, including 2D X-ray micro-imaging techniques and 3D X-ray tomography. Using X-ray micro-imaging techniques, we non-destructively monitored the inner *M. pudica* structures at a high resolution^17^,^18^. This study brings us one step closer to understanding the *M. pudica* motile mechanism.

## Results

### Tracking the *M. pudica* movements

**Figure 1:**
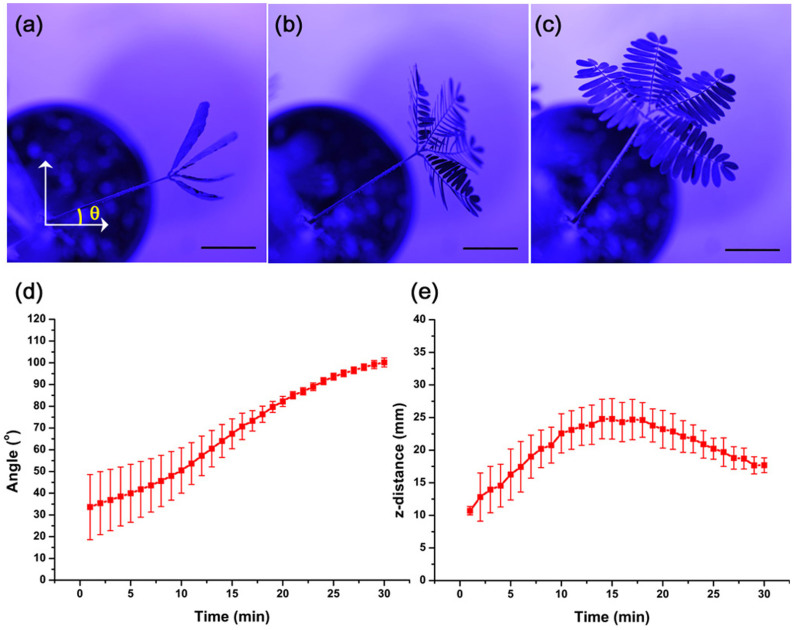
*M. pudica* motion in response to an external stimulus. *M. pudica* rotates its pulvinus and opens its leaves in response to 430 nm LED light. Top view images of *M. pudica* at the angles (*θ*) 23.2° (a), 33.9° (b) and 55.2° (c). The angles (d) and z-distance (e) of the pulvinus change with time lapsed after LED exposure. The error bars indicate the standard error (*n* = 3). Scale bar, 20 mm.

Without light, the *M. pudica* leaves are folded, and the pulvinus descends (Fig. 1a). In response to a 430 nm light-emitting diode (LED (PGL-E15, PARUS, Cheonan, Korea)), the leaves begin to unfold (Fig. 1b) and become completely unfolded in 30 minutes (Fig. 1c). Through morphological changes of the leaves, the pulvinus moves its body towards a light source. The bending angle between the initial and final positions of the pulvinus is 67.0° (Fig. 1d). In addition, the pulvinus elevates the z-position of the petiole 14 mm upward and then descends 7.1 mm downward (Fig. 1e). The moving trajectories show that the pulvinus can move its body vertically and horizontally.

Because the initial positions of the samples differ, the standard error bars are relatively large. Nevertheless, the error bar is substantially smaller at the final positions (Figs. 1d and 1e), which implies that the samples are at similar positions in the end. Thus, *M. pudica* can move omnidirectionally depending on the given environmental conditions.

### 3D pulvinus structural changes

Xylem vessel elements, which are dead cells, are connected to one another^19^,^20^. In addition, the xylem vessels cell walls contain lignin. Because lignin is firm and inflexible, the cell walls can maintain the vessel structures under a high negative hydrostatic pressure^21^,^22^. Most of the lignified elements have low flexibility; therefore, a snapping break motion is induced when they are forced to bend. Similar to other plants, the *M. pudica* stem has a strong and sustainable xylem. However, interestingly, the *M. pudica* pulvinus does not break off but only bends with high flexibility.

**Figure 2:**
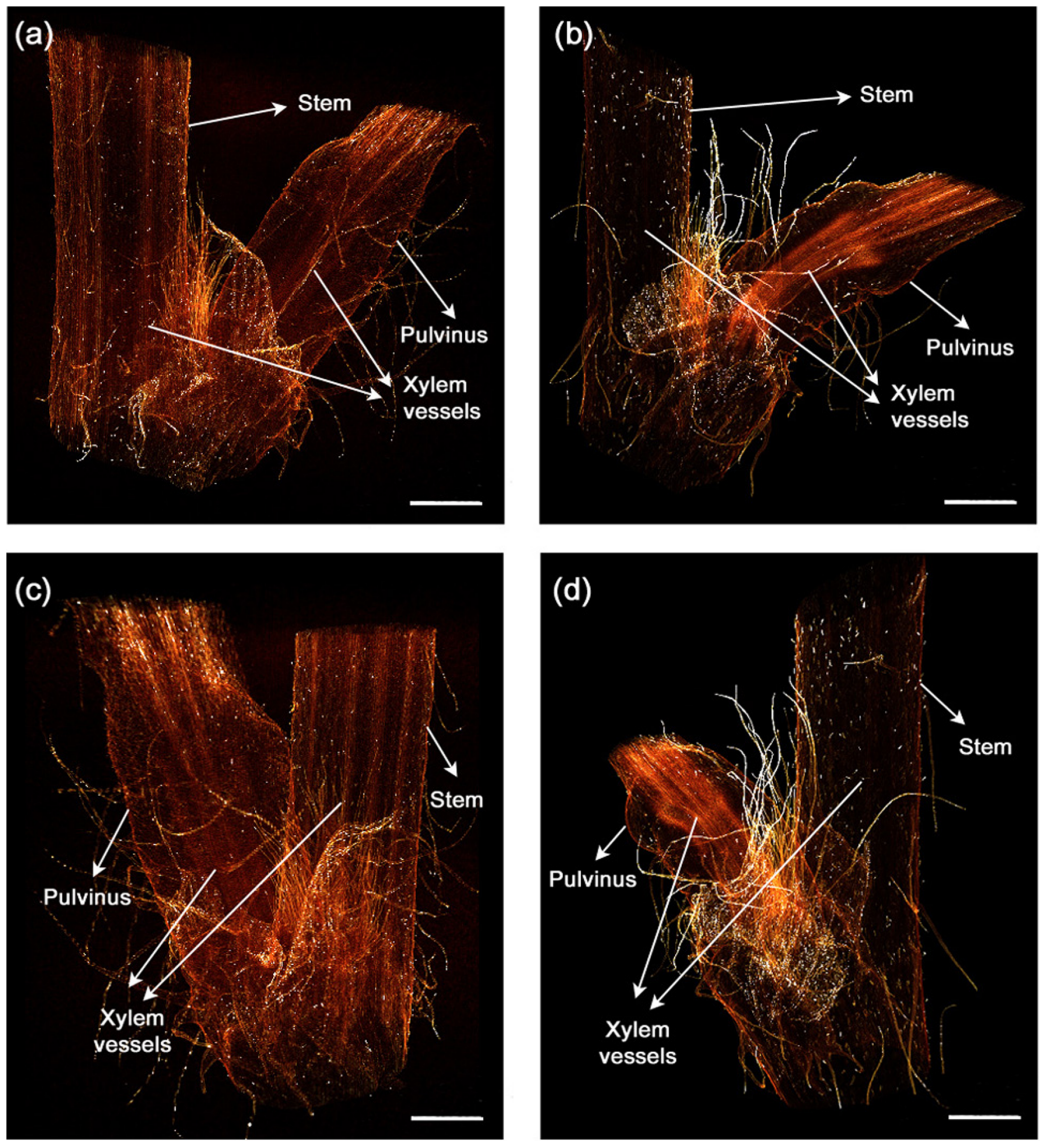
3D flexible structures of the straight and bending pulvinus. (a–d) X-ray tomograms show the internal morphological structure of the two different pulvini. 3D reconstructed images of the straight (a, c) and the bending (b, d) pulvini. The pulvinus reconstructed in a flank view (a, b) and a 130° rotated view around the vertical axis (c, d). The xylem vessels inside the pulvinus are straight or bent depending on the morphological condition. Scale bar, 2 mm.

Two distinguishable X-ray tomograms were compared (see Fig. 2) to investigate the morphological changes in the *M. pudica* bending xylem vessels. In all tomograms, the xylem vessels in the stem are straight. The xylem vessels in the straight pulvinus are upright and parallel to one another (Figs. 2a and 2c). However, they are bent in accordance with the pulvinus bending (Figs. 2b and 2d). These results demonstrate that the xylem vessels in the motile pulvinus have flexible structural properties that allow them to bend in all directions.

### Contraction and expansion of the pulvinus surface during a descending motion

**Figure 3:**
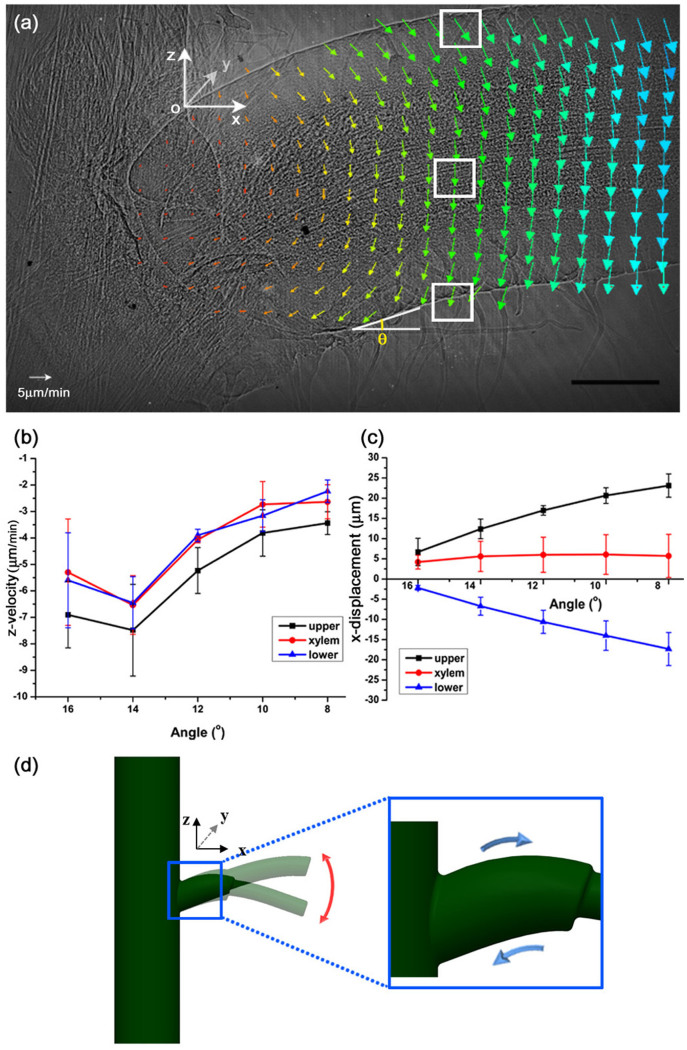
Expandable and contractible epidermis of the pulvinus. (a) Displacement of the epidermis and xylem of the pulvinus were obtained using PIV technique with X-ray microscopic images. A reference point O was set at the upper part of the pulvinus near the stem. The bending angle (*θ*) at the lower pulvinus depicted in (a) indicates the state of the bending motion. The upper epidermis moves in a positive direction, whereas the lower epidermis moves in a negative direction along the x-axis. (b) The z-directional velocities of the upper and lower epidermis and the xylems in the ROIs (white boxes) show a similar trend. (c) Their x-directional displacements exhibit different trend as the angle *θ* decreases. (d) A schematic diagram of the upper and lower epidermis of the pulvinus, which expands and contracts during bending. Error bars indicate standard error (*n* = 3). Scale bar, 500 μm.

Structural variations of the moving pulvinus were observed via X-ray microscopy (Fig. 3). The bending angle (*θ*) of the pulvinus decreased from 16.0° (0.3 mm^−1^) to 8.0° (0.1 mm^−1^). We analyzed displacement of the three parts of the pulvinus, including the upper epidermis, lower epidermis and xylem, during the descending motion in a 3D Cartesian coordinate system (Fig. 3a). Because this experiment did not include horizontal motion, the y-component was ignored. The three parts move downward and their displacement patterns are almost the same along the z-axis (Fig. 3). The velocity vectors of the moving pulvinus were obtained by applying a particle image velocimetry (PIV) algorithm to the X-ray images which were consecutively collected. The vectors are represented as arrows in Figure 3a. The z-direction velocity gradients in the region of interest (ROI; white boxes in Fig. 3a) for the three parts are shown in Figure 3b. The gradients exhibit similar trends in accordance with the bending angle.

The xylem vectors only contain z-components (Figs. 3b and c). However, the vectors in the upper and lower pulvinus have positive and negative x-components, respectively (Fig. 3a), which indicates that the x-directional displacements in the upper and lower epidermis move in opposite directions (Fig. 3c). As the pulvinus descended from *θ* = 16.0° to 8.0°, the particular upper epidermis was displaced by approximately 16.4 μm in the positive x-direction, whereas the particular lower epidermis was displaced by approximately (−) 15.2 μm in the negative x-direction. As shown in Figure 3d, to obtain the positive x-directional displacement, the upper epidermis must expand its surface area. On the other hand, the lower epidermis has a negative x-displacement, which indicates a contracting force (Fig. 3d). Based on these result, we concluded that expansion and contraction of the epidermis were simultaneous for the descending motion, even in the same pulvinus.

### Wrinkled structure of the pulvinus

The opposite directions of motion for the upper and lower parts of the epidermis were discussed above, but a question remains. How do the upper and lower epidermises expand/contract their surface areas? This deformable epidermis is explained by the wrinkle structure.

**Figure 4:**
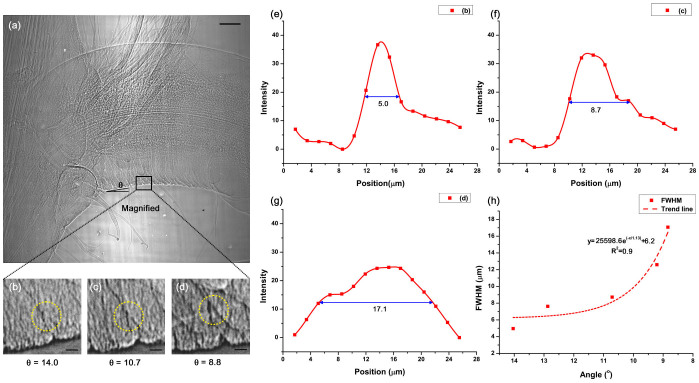
Spread of wrinkles on the pulvinus surface. (a) A typical X-ray image showing a large number of tiny wrinkles on the surface of the pulvinus. (b–d) As the angle (*θ*), which is depicted in the pulvinus bending motion, decreases, the tiny wrinkles spread out. Inverse images in the ROI at the angles (b) 14.0°, (c) 10.7° and (d) 8.8° were magnified to clearly show the wrinkles in 2D X-ray images. (e–g) The image intensity profiles were measured around the wrinkles (dashed circles in b–d) and (h) their FWHMs were calculated. The FWHM values exponentially increased with an increasing pulvinus bending angle. The scale bars in (a) and (b–d) indicate 1 mm and 100 μm, respectively.

Wrinkles can considerably increase the surface area; thus, human fingers and elbows have numerous wrinkles^23^,^24^. Likewise, the pulvinus surface has plenty of tiny wrinkles (Fig. 4a). This rugose structure provides extra surface area for expansion or contraction.

The wrinkles on the lower parts of the pulvinus that are used for bending motions were visualized using X-ray microscopy. The shrinking pulvinus decreases the surface area by folding its epidermis and forming wrinkles. As the epidermis folds, the image intensity of the wrinkles clearly increases (Fig. 4a). Figure 4e shows the intensity variation at a local region near a wrinkle (white box in Fig. 4a). The full width at half maximum (FWHM) value 5.0 μm was calculated from the intensity profile when the pulvinus angle was 14.0° (0.3 mm^−1^) (Fig. 4b). The wrinkle is more apparent, and the FWHM is 8.7 μm at a 10.7° (0.2 mm^−1^) bending angle compared with wrinkles at a 14.0° bending angle (Figs. 4c, f). Moreover, the wrinkle appears thicker (Fig. 4d), and the FWHM is 17.1 μm (Fig. 4g) at a 8.8° (0.2 mm^−1^) bending angle, which is almost 3.4 times wider than at 14.0° (Fig. 4e). The bending motion accompanies the reduced surface area at the lower epidermis, and the wrinkle reduces the surface area. Thus, the increased bending angle induces a deeper wrinkle.

Figure 4h shows that the FWHM values exponentially increase based on the smaller pulvinus bending angle. The *R^2^* of the fitting curve is 0.9, which indicates that the regression curve is consistent with the FWHM values.

### Xylem structure along the pulvinus

The majority of previous studies on the seismonastic reaction of *M. pudica* have focused on motor cells and water transport^6^,^7^,^8^,^9^,^10^,^11^,^12^,^13^,^14^. One of the most remarkable results is the immediate disappearance of water in the lower part of the pulvinus as it leans down^25^. This result implies that the *M. pudica* motion is deeply related to water transport. Because xylem vessels are passages for water transport, the pulvinus xylem vessels are worth studying.

**Figure 5:**
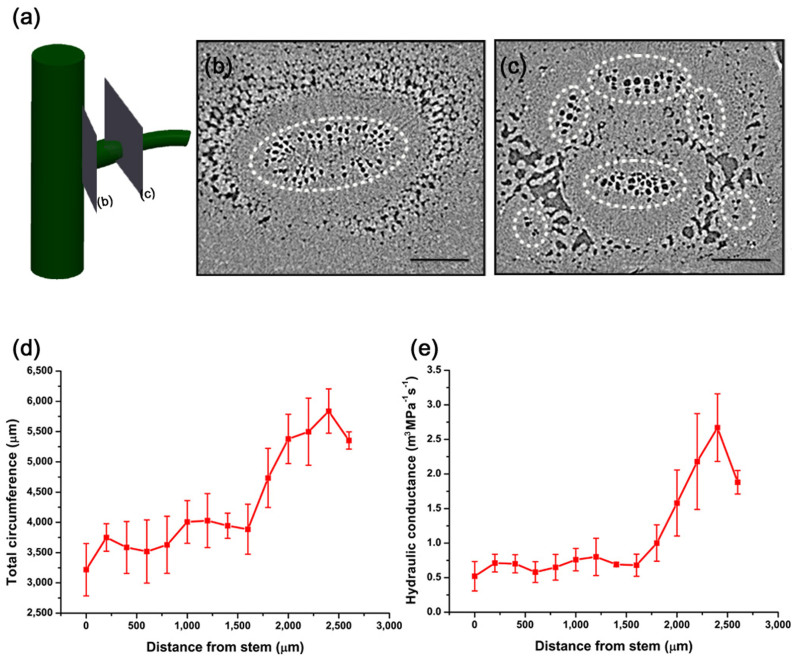
Morphological characteristics of xylem vessels in the pulvinus. Cross-sectional X-ray images of the pulvinus branched from the main stem to the petiole side. (a) A schematic diagram shows the cross-sectional imaging positions 200 μm (b) and 2600 μm (c) from the main stem. In the pulvinus, one xylem bundle (b) and six reorganized, independent bundles were observed (c). A xylem bundle in the cross-sectional images is depicted as a white dashed circle. (d) The total circumference and (e) hydraulic conductance of the xylem vessels vary with the distance from the main stem. The circumference and hydraulic conductance rapidly increase beyond a specific point due to larger surface area and greater number of xylem vessels. Error bars indicate standard error (*n* = 3). Scale bar, 200 μm.

The *M. pudica* motile pulvinus xylem networks differ from the immobile stems of *M. pudica*, which have simple parallel structures (Fig. 2). The cross-sectional X-ray images of a pulvinus that branches from the stem to the petiole side are presented in Figure 5a. Similar to other vascular plants, the xylem vessels of *M. pudica* form a circular xylem bundle in the cross-section (b) 200 μm away from the stem (Fig. 5b). This bundle is depicted as a white, dashed circle. However, six independent bundles are shown in the cross-section (c) near the petiole (2600 μm away from the stem; Fig. 5c). These bundles are spread along the inner side of the pulvinus and are surrounded by lignified walls and air spaces.

This dramatic change in reorganization occurs with a rapid increase in the number and surface area of xylem vessels, which yields the sharp increase in xylem circumference and hydraulic conductance of the pulvinus (Figs. 5d and 5e). The total circumference of the xylem bundles in Figure 5c is 1.5-fold greater than the single bundle in Figure 5b. Considering the vessel diameter and number of conduits, hydraulic conductance was determined, which is the ability to allow water flow^26^. The hydraulic conductance of the pulvinus depicted in Figure 5c is 5.1-fold greater than in Figure 5b. Thus, a steep rise in the hydraulic conductance is attributed to an increase in the vessel diameter and number of conduits because the hydraulic conductance is proportional to the sum of the fourth power of the xylem radii (Eq. 3).

These increases imply that the xylem vessels' large circumference and high hydraulic conductance are intimately related to enhanced water exchange in the pulvinus. The xylem vessels have more opportunities for water exchange with cells through a greater surface area under high hydraulic conductance. Therefore, the pulvinus provides more opportunities for water transport compared with the stem.

## Discussion

In this study, the structural characteristics were observed using an X-ray microscopy technique with high spatial resolution to prevent the samples from damage during preparation. This technique is advantageous over conventional microscopy. Since vascular plant cells are surrounded by lignified dead cells, their inner morphological structure is not easily detected using optical imaging methods. Therefore, the sectional structure of *M. pudica* could not be investigated previously until a series of chemical treatments were performed^27^,^28^.

Absorption, refraction, and diffraction of imaging techniques have been utilized to obtain absorption-contrast and/or phase-contrast X-ray images^29^. Combined with X-ray microscopy, these techniques can provide sufficiently high resolution to observe the internal structures of *M.* *pudica* in detail^17^. The X-ray micro-imaging techniques non-destructively monitor the temporal variation of xylem vessels under *in vivo* conditions^18^. Therefore, we studied the structural changes in the pulvinus of moving *M. pudica* in real-time.

The pulvinus movements and its structural advantages for bending were investigated experimentally using X-ray microscopy. In response to 430nm LED light, the *M. pudica* leaves are open widely, and the pulvinus reacts by performing vertical and horizontal motions. The *M. pudica* pulvinus exhibits distinctive responses that depend on different types of stimuli^20^. These responsive motions without a snapping break may be due to the pulvinus structural characteristics. Owing to the bendable features of its xylem vessels and wrinkled epidermis, the pulvinus works as a flexor or extensor during motion. In addition, reorganization of the xylem vessels yields in high hydraulic conductance that facilitates water transport and considerably increases the cell contact area. These structural advantages and unique pulvinus feature presumably contribute to high motility in *M. pudica*.

Nevertheless, the *M. pudica* motile mechanism has not been thoroughly established in detail. Water transport in the pulvinus and leaves has been considered a primary driving force for *M. pudica* movement^30^. In fact, a motile plant may have highly efficient hydraulic systems for rapid water transport through xylem vessels. These unidentified hydraulic systems, including the conduit for water transport and correlation between the water flow as well as pulvinus motion should be studied to fully grasp the motile mechanism of *M. pudica*.

## Methods

### Plant

Well grown and healthy *M. pudica* samples were used for this experiment. The samples were grown in an air-conditioned room, which was maintained at 25°C and 70% humidity with the photoperiod 8 h:16 h light:dark. We used LED lamps (PGL-E15, PARUS, Cheonan, Korea) with a 3:6:3 ratio of white: 430 nm: 660 nm. The light intensity on the plant leaf surface was 500 mmol/s/m^2^.

### Tracing the movement of *M. pudica* in response to light

Before exposure to 430 nm LED light, the plant samples were placed in a dark room for an hour. The light source was initially positioned opposite the direction that the leaves were facing to induce rotation in the pulvinus. The plants were then exposed to light for 30 minutes. The dynamic motion of the pulvinus was recorded from a top view using a digital camera (Nikon D700, Japan) at 1 frame/min. The angular displacement and length variation of petioles at an orthogonal projection were measured using the Image J program (National Institute of Health, USA). Since the actual petiole length in three dimensions was nearly constant during the entire measurement, the height variation (z-distance) can be calculated using the actual and measured lengths of the petiole using the following equation. 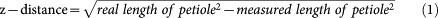


### X-ray tomography

The 3D morphological structures of the samples were observed using X-ray micro computed tomography (CT) at the 6D beamline of the Pohang Accelerator Laboratory (PAL). A mechanical shutter was employed to block the X-ray beam except during image acquisition to minimize photo thermal damage to the sample. The distance from the plant sample to the camera was 30 cm to enhance the phase contrast effect. The X-ray images were collected using a charge-coupled device (CCD) camera with 4008 × 2672 pixel resolution (Vieworks VM-11M, Korea) at 7 mm × 4.6 mm field of view (FOV). The spatial resolution based on camera the pixel size with a ×5 objective lens was 1.7 μm/pixel. The sample was fixed to a sample holder when the stage was rotated from 0° to 180° at 0.5° intervals. The sample was frozen with a continuous supply of liquid nitrogen in a cryo-system to prevent structural modification during acquisition of the X-ray images.

Each acquired image was reduced to 2004 × 1336 pixels using ×2 binning with Octopus software (inCT, Belgium) for rapid data processing. Erroneous spots in the X-ray images were removed, and a tomogram was reconstructed using Octopus software. The reconstructed images were then analyzed using Amira software (Visualization Science Group, USA).

### X-ray micrography

2D serial images were collected using X-ray microscopy at the PAL 6C beamline. A 2 mm graphite attenuator and 1.5 mm-thick silicon wafers were positioned in the X-ray beam pathway to minimize the photo thermal damage to the samples. The FOV was 4 mm × 3.5 mm and the distance from the plant sample to the camera was 20 cm. The X-ray images were consecutively collected using a sCMOS camera (Andor Zyla, UK) with 2560 × 2160 pixels at 1 frame/min. The spatial resolution was 1.6 μm/pixel, which was based on the pixel size of this camera with a ×4 objective lens. A 430 nm LED lamp was installed next to the detector to stimulate the *M. pudica*. The sample stem was fixed to the sample holder.

### Measurement of structural changes

Images of the epidermis displacement were collected using X-ray microscopy; the images were analyzed using a PIV technique based on a cross-correlation algorithm. Two consecutive images were recorded with a 2 minute time interval. Each image was divided into 64 × 64 pixel interrogation windows with a 50% overlap. A recursive correlation algorithm was applied to the interrogation windows to enhance the measurement accuracy. The representative displacement between the interrogation windows was estimated by detecting the peak position. This peak detection procedure was repeated for all windows to determine the spatial distribution of structural displacement. A reference point O (Fig. 3a) was set at the upper side of the pulvinus near the stem to obtain displacement information on the epidermis structure using regular grids. Each ROI centroid was located at x = 1024 μm. The displacement was investigated at the top, bottom and middle of the pulvinus, which correspond to the upper epidermis, xylem bundles and lower epidermis, respectively. Considering that the pulvinus moves mainly in the z direction, the z-directional velocity and x-directional displacement were analyzed. The mean values of three samples were statistically determined at five angles to discern the general motion trend.

### Measuring the morphological variation in the wrinkles

The image intensity spatial distributions were measured to investigate wrinkle spreading. First, the 2D X-ray images were precisely rotated using a digital image-processing technique to horizontally orient the lower curvature of the pulvinus near the particular wrinkle. The middle of a wrinkle was designated the reference point. Based on this reference point, the image intensities in the ROI for 15 × 2 pixels were averaged along the vertical direction. The intensity data were subtracted by the minimum value. These intensity data were depicted using a graph through a piecewise cubic hermite interpolating polynomial (PCHIP) method. The FWHM of the intensity distributions were estimated using regression analysis.

### Evaluating the contact area of cells and hydraulic conductance

In the reconstructed images, the number of xylem vessels and their surface areas were quantitatively analyzed using the Image J program (National Institutes of Health, USA). Each radius of each xylem vessel was calculated from its surface area. The circumference of the xylem vessel was calculated using the estimated radius. The volumetric flow rate (*Q*) was estimated using the Hagen-Poiseuille law. 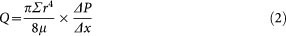
Here, *ΔP* is the pressure difference between the two ends of a xylem vessel. The hydraulic conductance *k_h_*(m^3^Pa^−1^s^−1^) was determined using the following formula. 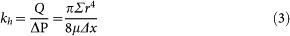
These equations were established based on the characteristics of the laminar flow in ideal, long horizontal and constant-diameter tubes^19^,^31^. The *μ*(Pa·s) denotes the dynamic fluid viscosity, whereas r and *Δx* are the internal radius and length of the tube, respectively. The working fluid was assumed to be water at 20°C with a dynamic viscosity *μ* of 1 × 10^−3^ Pa·s.
